# Whole transcriptome analysis of the hippocampus: toward a molecular portrait of epileptogenesis

**DOI:** 10.1186/1471-2164-11-230

**Published:** 2010-04-08

**Authors:** Oswaldo K Okamoto, Luciana Janjoppi, Felipe M Bonone, Aline P Pansani, Alexandre V da Silva, Fúlvio A Scorza, Esper A Cavalheiro

**Affiliations:** 1 Disciplina de Neurologia Experimental, Departamento de Neurologia e Neurocirurgia, Universidade Federal de São Paulo; 2 Departamento de Biociências, Universidade Federal de São Paulo

## Abstract

**Background:**

Uncovering the molecular mechanisms involved in epileptogenesis is critical to better understand the physiopathology of epilepsies and to help develop new therapeutic strategies for this prevalent and severe neurological condition that affects millions of people worldwide.

**Results:**

Changes in the transcriptome of hippocampal cells from rats subjected to the pilocarpine model of epilepsy were evaluated by microarrays covering 34,000 transcripts representing all annotated rat genes to date. Using such genome-wide approach, differential expression of nearly 1,400 genes was detected during the course of epileptogenesis, from the early events post *status epilepticus (SE) *to the onset of recurrent spontaneous seizures. Most of these genes are novel and displayed an up-regulation after *SE*. Noteworthy, a group of 128 genes was found consistently hyper-expressed throughout epileptogenesis, indicating stable modulation of the p38MAPK, Jak-STAT, PI3K, and mTOR signaling pathways. In particular, up-regulation of genes from the TGF-beta and IGF-1 signaling pathways, with opposite effects on neurogenesis, correlate with the physiopathological changes reported in humans.

**Conclusions:**

A consistent regulation of genes functioning in intracellular signal transduction regulating neurogenesis have been identified during epileptogenesis, some of which with parallel expression patterns reported in patients with epilepsy, strengthening the link between these processes and development of epilepsy. These findings reveal dynamic molecular changes occurring in the hippocampus that may serve as a starting point for designing alternative therapeutic strategies to prevent the development of epilepsy after acquired brain insults.

## Background

Chronic brain disorders have a profound impact on life quality since most of them are associated with cognitive impairment, and disturbance of personality or behavior. With the tendency of global population aging, the incidence of people living with such disabilities will dramatically increase in the next decades. Epilepsy is one example of prevalent and severe neurological condition, affecting approximately 50 million people worldwide [1].

Epidemiological studies reveal that about 20 to 30% of patients with epilepsy are refractory to the currently available therapies and continue to have seizures throughout their lives [2]. Temporal lobe epilepsy (TLE), which is characterized by atrophy of mesial temporal structures and hippocampal sclerosis, is the most frequent form of partial epilepsy and also the most common form of drug-refractory epilepsy [3].

The mechanisms of action of most clinically used drugs in human established epilepsies are based upon the synchronized neuronal activity and unbalance between inhibitory and excitatory neurotransmission, which are common features linked to the pathogenesis of epilepsy [4]. In that sense, voltage-gated ion channels, gabaergic, and glutamatergic systems are the classic therapeutic targets. However, these drugs act to restrain epileptic seizures in already established epilepsies rather than preventing the development of epilepsy after acquired brain insults.

Uncovering the molecular mechanisms involved in epileptogenesis is critical to understand the physiopathology of epilepsies and to help develop new therapeutic strategies based on drugs with anti-epileptogenic activity. The identification of potential therapeutic targets, however, should be facilitated by the knowledge of genes, proteins, and signaling pathways altered during the different stages of epilepsy development.

In the post-genomic era, DNA microarrays are being widely used as an experimental tool to monitor changes in gene expression levels in different pathologies. In epilepsy, the clinical implications of the microarray technology are illustrated in a few recent publications in the literature [5-9]. One drawback of such approach in humans, however, is that the experimental design is not trivial due to the lack of appropriate control samples of healthy brain tissue. Furthermore, surgical specimens often available for study do not allow correlation with the early stages of the disease. Alternatively, key molecular alterations during epileptogenesis can be examined in animals subjected to classical models of experimental epilepsy. Several well-characterized models have been described in the last decades and in many ways they mimic complex partial seizures observed in patients with TLE [10].

Recent reports of gene expression profiling are available for some of these animal models [11-15], albeit with partial transcriptome coverage and time points more closely related with responses to either *status epilepticus *(*SE*) or cumulative chronic spontaneous seizures. A comprehensive gene expression profiling designed for the study of epileptogenic process, from the early molecular changes induced by hippocampal injury to the onset of epilepsy, is still lacking.

In this work, we performed a genome wide analysis of genes differentially expressed during epileptogenesis. Virtually, all possible changes in the rat transcriptome were monitored at distinct time points corresponding to the latent to chronic phase transition of the pilocarpine model of epilepsy, one the most extensively studied chemically induced model of TLE [16-18]. Genes identified as being differentially expressed were classified based on their respective biological functions to envisage processes and pathways likely implicated in epileptogenesis. Genes stably up-regulated and working in a concerted fashion were identified, some of which displaying a parallel expression pattern in humans with epilepsy, suggesting their possible value as targets for therapy.

## Methods

### Animal model

Male adult Wistar rats (200-250 g) were used throughout the study. Animals were housed under standard controlled conditions (7:00 AM/7:00 P.M. light/dark cycle; 20-22°C; 45-55% humidity) with food and water *ad libitum*. All efforts were made to minimize animal suffering following the proposal of International Ethical Guideline for Biomedical Research (CIOMS/OMS, 1985). The study was approved by the Ethics Committee for animal research of the Federal University of São Paulo, Brazil (CEP 2053/07).

Animals (n = 5 per experimental group) were subjected to the pilocarpine model of epilepsy [19,20]. Briefly, rats were pre-treated with a subcutaneous injection of scopolamine methylnitrate (1 mg/kg, to minimize peripheral cholinergic effects), followed by a single dose of pilocarpine (350 mg/kg, dissolved in saline) (Sigma Chemical, USA) injected intraperitoneally. Control animals received the same amount of saline only. After 4 h of *SE*, pilocarpine-treated rats (Pilo) received diazepam (8 mg/kg i.p.) to block behavioral and electrographic seizures. Occurrence of spontaneous seizures was detected by continued video monitoring of animals.

### Microarray hybridization

Gene expression profiling was analyzed in hippocampi of rats treated with pilocarpine at different times during the course of epileptogenesis: 3 days post-*SE *(3 D), 7 days post-*SE *(7 D), and immediately (up to 12 h) after the first spontaneous seizure (Chronic). All three pilocarpine subgroups had a corresponding age-matched control group (saline-treated rats), from which normal hippocampi samples were obtained at the same experimental time points. Rats were killed by decapitation and the hippocampi were removed at 4°C within a period no longer than three minutes. Brain tissues were snap frozen in liquid nitrogen and stored at -80°C until RNA extraction with the RNeasy kit (Qiagen), following the manufacturer's protocol. Evaluation of RNA quantification and purification was carried out by measuring absorbance at 260 and 280 nm. A260/A280 ratios in the range of 1.8-2.0 were considered satisfactory for purity standards. Denaturing agarose gel electrophoresis was used to assess the quality of RNA samples.

Independent microarray hybridizations were carried out for each sample (n = 5, per experimental group) with oligonucleotide microarrays covering 34,000 transcripts representing all known and predictive genes of the rat genome (CodeLink™ Rat Whole Genome Bioarrays, GE Healthcare), following the manufacturer's protocol. Detailed descriptions of the Bioarray platform and expression data are publicly available, according to MIAME guidelines, at the GEO database under the accession numbers GPL2896 and GSE14763. Briefly, total RNA was extracted with RNeasy spin columns and treated with RNAse free-DNAse (Qiagen). One microgram of total RNA was reverse transcribed in the presence of T7- oligo dT primer. The resulting cDNA was used for *in vitro *transcription reaction using T7 RNA polymerase and biotinylated dUTP. Ten micrograms of target cRNA was fragmented at 94°C for 20 min and hybridized to the bioarrays at 37°C for 18 h, under 300 rpm agitation. After staining with streptavidin-conjugated Cyanine-5 dye, the slides were washed and fluorescence was measured using an Axon GenePix 4000B Scanner (Axon Instruments Inc).

### Gene Expression measurement

The fluorescent images were captured using GenePix Pro v.4.1 (Axon Instruments Inc) and the light intensities were quantified, corrected for background noise, and normalized with the CodeLink™ Expression Analysis v4.1. Spots with background contamination, shape irregularity, or pixel saturation were filtered out. Only spots flagged as "good" (G) or "low" (L) were considered in the subsequent analysis. Since the CodeLink™ system is a one-color platform, we grouped the meaningful comparisons in pairs to form ratios, as usual in two-color co-hybridized microarray platforms. Differentially expressed genes were identified by comparing the fluorescence values of a given spot (transcript) in samples from pilocarpine-treated rats vs. pair-matched control rats, using an intensity-dependent dynamic cutoff with 99% credibility level. Genes displaying expression changes equal or higher than two fold (P ≤ 0.05) were included in the analysis. These differentially expressed genes were grouped by unsupervised hierarchical clustering using correlation and average linkage metrics as described elsewhere [21].

### Gene Enrichment and Functional Annotation Analysis

Given the lists of differentially expressed genes, we performed an ontology term enrichment analysis using the DAVID 2.1 tool to find gene functional classes specifically associated with those lists [22]. In such analysis, the statistical association between being differentially expressed and belonging to a given category is accessed by the Fisher Exact test. The ontologies used were those defined by the KEGG database of metabolic pathways [23] and by the Gene Ontology Consortium [24]. The probe-to-GO and the probe-to-KEGG mapping were established based on the official annotation provided by the manufacturer.

### Real-time PCR quantification

Gene expression was quantified in hippocampi of rats treated either with saline or pilocarpine, at different times during the course of epileptogenesis (n = 5 per experimental group). One microgram of total RNA was used to synthesize cDNA by extension with oligo-dT primers and 200 U of Superscript II Reverse Transcriptase (Invitrogen Life Technologies). Quantitative reverse-transcription polymerase chain reaction was performed in a ABI 7500 Real-Time PCR System platform (Applied Biosystems) by the Sybr-Green approach (Platinum SYBR Green qPCR SuperMix-UDG, Invitrogen Life Technologies), using ROX as passive reference dye. All samples were analyzed in the same plate, in a single PCR run, and under identical conditions. Amplification specificity was assessed by dissociation curve analysis. Normalization of quantitative data was based on the expression of the housekeeping gene glyceraldehyde-3-phosphate dehydrogenase (GAPDH). Quantification was based on 2^-ΔΔCT ^method, using corresponding age-matched control samples as calibrators. Amplification reactions contained 2 μL of 1:10 diluted cDNA and 10 pmoles of each primer. Basic amplification cycle suggested by the manufacturer was used, with an annealing temperature of 60°C. Primer sequences were obtained elsewhere: *NESTIN *FOR 5' AGCAACTGGCACACCTCAAG 3', *NESTIN *REV 5' GGTGTCTGCAAGCGAAAGTTC 3', *GFAP *FOR 5' CTCAGTACGAGGCAGTGGCC 3', *GFAP *REV 5' CGGGAAGCAACGTCTGTGA 3' [25], *IGF1 *FOR 5' CACAGGCTATGGCTCCAGCAT 3', *IGF1 *REV 5' TCTCCAGCCTCCTCAGATCACA 3' [26], *CDK1 *FOR 5' CGGTTGACATCTGGAGCATA 3', *CDK1 *REV 5' GCATTTTCGAGAGCAAGTCC 3', *p18(INK4c) *FOR 5' ACCGAACTGGTTTTGCTGTC 3', *p18(INK4c) *REV 5' GGGCAGGTTCCCTTCATTAT 3' [27], *TGFbeta1 *FOR 5' GAGAGCCCTGGATACCAACTACTG 3', *TGFbeta1 *REV 5' GTGTGTCCAGGCTCCAAATGTAG 3' [28], GAPDH FOR 5' GAACCTGCCGTGGGTAGAG 3', GAPDH REV 5' AGGTCGGTGTGAACGGATTTG 3' [29].

### Statistical Analyses

Microarray data: statistically significant differences among treatments were determined by the Student's *t *test and the Fisher Exact test. All conclusions are based on at least 5% level of significance (*P *≤ 0.05). Real-time PCR data: analysis was performed with the GraphPad Prism software, v.3.00 for Windows (San Diego, CA, USA). Data were analyzed by Two-way ANOVA, Treatment (2) × Time (3), with Bonferroni as *post-hoc *test, since there was a normal distribution. Significance was established at the P ≤ 0.05 level.

## Results

### Latent period of the pilocarpine model is marked by dynamic changes at the molecular level

As extensively described by Turski et al., 1983 [19], a single high dose of pilocarpine, a potent muscarinic cholinergic agonist originally isolated from the leaves of South American shrubs, induced sequential behavioral changes characteristic of sustained epileptic activity. Few minutes after pilocarpine administration, the animals exhibited stereotypical oral and mastigatory movements, hypokinesia, salivation, chewing, sniffing-head, tremor, and partial or generalized limbic seizures that evolved to *SE*. The mortality within the first 24 h of pilocarpine administration was around 30%. Only those animals that achieved *SE *and survived were included in the study (n = 5 per experimental group). This acute phase was followed by a latent period during which the animals were initially comatose or unresponsive to their environment due to *SE *and progressively returned to normal behavior, usually within three days post-*SE*. This period is also described as a "silent" period due to the absence o behavioral and electrographic evidences of seizures. Spontaneous and recurrent seizures typically characterized by facial automatism, forelimb clonus, and rearing followed by loss of postural control and generalized clonic seizures lasting 25-35 sec. tend to occur later on, being a hallmark of the chronic period of the pilocarpine model. Time to first spontaneous seizure ranged from nine to 17 days. The latent period of the pilocarpine model of epilepsy is known to vary among animals and our results are in agreement with the literature [16].

**Figure 1:**
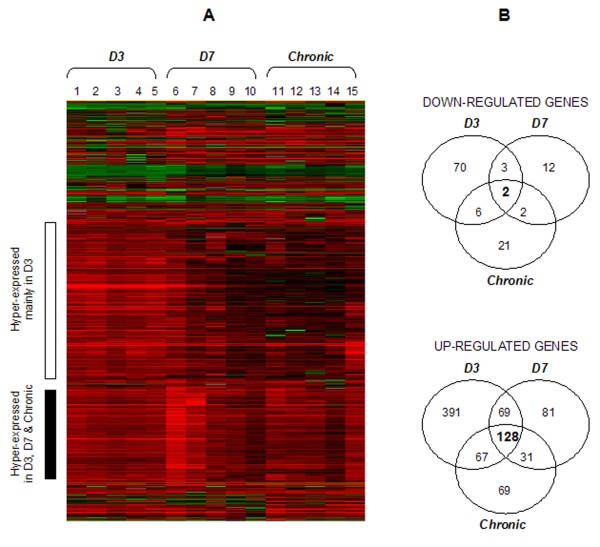
**Gene expression profiling during epileptogenesis**. (**A**) Hierarchical clustering of hippocampal genes displaying greater than 2-fold changes in expression following *SE *(P ≤ 0.05). Each row: relative expression of a single gene; each column: a biological sample; red: hyper-expression; green: hypo-expression. Several genes exhibited a consistent hyper-expression throughout the time points analyzed (black bar cluster) while others were mainly hyper-expressed at D3 (white bar cluster). (**B**) Venn diagram discriminating the amount of genes differentially expressed three days post-SE (D3), seven days post-*SE *(D7), and immediately after the first spontaneous seizure (Chronic).

Although clinically silent, the period spanning *SE *and the beginning of spontaneous seizure activity is critical to evaluate changes that might be relevant to epileptogenesis. Therefore, a whole transcriptome analysis was performed with the purpose of identifying genes differentially expressed after pilocarpine-induced *SE*. The starting total RNA used in the hybridizations was extracted from hippocampi at different times during the course of epileptogenesis. After a typical microarray-based analysis (Figure 1), alterations in the expression of almost 1,400 genes were detected, suggesting an intense molecular activity during the latent to chronic phase transition of the pilocarpine model. A total of 736, 328, and 326 genes were found differentially expressed three days post-SE (D3), seven days post-SE (D7), and after the first spontaneous seizure (Chronic), respectively. Noteworthy, the vast majority of these differentially expressed genes (90%) had their expression up-regulated after *SE*, while the remainder 10% corresponded to hypo-expressed genes at the same time points analyzed (D3 = 81, D7 = 19, Chronic = 31).

### Pilocarpine-induced *SE *promotes constitutive changes in gene expression

Among the 1,400 differentially expressed genes, of particular interest was the finding of a large group of 128 genes commonly over-expressed in D3, D7, and Chronic groups, indicative of a stable gene up-regulation throughout the latent to chronic phase transition of the pilocarpine model. As depicted in the Venn diagram of figure 1B, this amount of commonly over-expressed genes is higher than the number of genes exclusively up-regulated at each of the respective time points, with the exception of D3. In contrast, among the down-regulated genes, only two genes were found simultaneously hypo-expressed at all time points analyzed and, to date, no biological functions have been described for them.

### Biological processes and signaling pathways associated with epileptogenesis

When functionally classifying genes based on the Gene Ontology Consortium terminology, a myriad of biological processes could be related to the genes affected by *SE*. Since the overall representation of genes and corresponding annotated functions in the entire rat genome may confer a bias in this type of functional classification and because a single gene may belong to multiple functional categories, one major challenge is to distinguish processes and functions specifically associated with epileptogenesis from those found due to random chance. Therefore, a statistical test was applied to circumvent this issue and identify functional categories over-represented among the genes regulated by *SE *in hippocampal cells.

**Figure 2:**
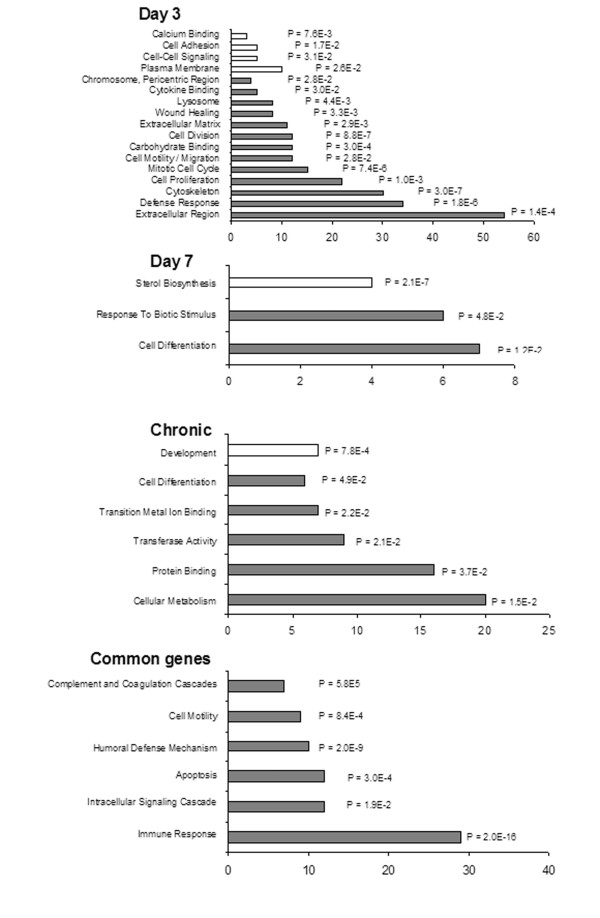
**Functional classification of genes differentially expressed during epileptogenesis**. Fisher Exact test was applied to identify functional categories over-represented among the genes regulated by *SE *in hippocampal cells, thereby ensuring statistical significance. D3 = three days after *SE*; D7 = seven days after SE; Chronic = immediately after the first spontaneous seizure; Common = genes found differentially expressed in all previous groups.

Figure 2 summarizes the number of enriched genes, their corresponding molecular functions, and respective *P*-values for functional association. Such classification strategy revealed that the subset of 128 genes commonly up-regulated in D3, D7, and chronic groups were particularly enriched in genes functioning in immune response (including humoral defense mechanism and, more specifically, complement and coagulation cascades), cell motility, apoptosis, and intracellular signaling cascades. The differentially expressed genes and respective functional categories are depicted in table 1. Regarding signaling pathways, those corresponding to over- and hypo- expressed genes during all experimental times studied after *SE *are illustrated in table 2. From this later analysis, it can be noticed that MAPK, Jak-STAT, Phosphatidyl inositol, TGF-beta, and mTOR signaling pathways were found regulated in pilocarpine-treated animals throughout the epileptogenesis period evaluated.

**Table 1:** Genes commonly up-regulated throughout epileptogenesis, from the early events post-*SE *to the onset of recurrent spontaneous seizures.

Accession No.	Fold change			Description
		
	D3	D7	Chronic	
**Immune response**				
RN.72599	5,1	12,2	7,4	colony stimulating factor 1 receptor
RN.16195	3,7	4,0	3,2	caspase 11
RN.14655	9,6	5,6	4,1	integrin alpha l
RN.16643	4,8	4,2	3,1	fc receptor, igg, high affinity i
RN.2393	5,7	5,0	3,6	complement component 1, q subcomponent, gamma polypeptide
RN.6702	5,0	4,8	3,1	complement component 1, q subcomponent, beta polypeptide
RN.10089	4,7	4,2	3,8	cyclin-dependent kinase inhibitor 1a
RN.48861	5,2	4,8	3,6	vav 1 oncogene
RN.10748	2,9	3,9	5,5	cd4 antigen
RN.81052	2,7	4,5	4,5	complement component 4a
RN.5892	5,8	6,3	5,1	major histocompatibility complex, class ii, dm beta
RN.780	7,0	3,9	4,1	alpha-2-macroglobulin
RN.38575	3,2	3,8	3,5	neutrophil cytosolic factor 1
RN.100285	7,7	9,9	8,3	serine (or cysteine) peptidase inhibitor, clade g, member 1
RN.92197	4,7	6,5	5,9	linker for activation of t cells family, member 2
RN.3176	4,2	4,7	3,8	lymphocyte antigen 86 (predicted)
RN.101608	4,9	4,2	4,5	lymphocyte specific 1
RN.58420	3,1	2,6	2,7	similar to ikappab-zeta (predicted)
RN.16670	5,9	4,2	4,5	basic leucine zipper transcription factor, atf-like (predicted)
RN.98333	4,2	4,6	4,0	complement component 2
RN.42962	4,3	6,8	6,2	integrin beta 2
RN.105647	4,1	4,4	3,3	complement component 1, q subcomponent, alpha polypeptide
RN.37880	4,7	4,6	5,6	small inducible cytokine a4
RN.10330	6,7	4,8	5,9	cd8 antigen, beta chain
RN.10139	10,9	12,8	10,1	chemokine (c-c motif) ligand 3
RN.30043	4,8	5,7	4,9	tumor necrosis factor (ligand) superfamily, member 4
RN.3370	5,7	4,6	4,3	interferon gamma inducible protein 30
RN.90166	3,4	3,7	3,2	protein tyrosine phosphatase, receptor type, c
RN.33323	6,0	6,0	4,8	fc receptor, igg, low affinity iib
				
**Humoral Defense Mechanism**				
RN.105647	4,1	4,4	3,3	complement component 1, q subcomponent, alpha polypeptide
RN.81052	2,7	4,5	4,5	complement component 4a
RN.72599	5,1	12,2	7,4	colony stimulating factor 1 receptor
RN.100285	7,7	9,9	8,3	serine (or cysteine) peptidase inhibitor, clade g, member 1
RN.2393	5,7	5,0	3,6	complement component 1, q subcomponent, gamma polypeptide
RN.10139	10,9	12,8	10,1	chemokine (c-c motif) ligand 3
RN.16670	5,9	4,2	4,5	basic leucine zipper transcription factor, atf-like (predicted)
RN.6702	5,0	4,8	3,1	complement component 1, q subcomponent, beta polypeptide
RN.42962	4,3	6,8	6,2	integrin beta 2
RN.33323	6,0	6,0	4,8	fc receptor, igg, low affinity iib
				
**Complement and Coagulation Cascades**				
RN.81052	2,7	4,5	4,5	complement component 4a
RN.780	7,0	3,9	4,1	alpha-2-macroglobulin
RN.100285	7,7	9,9	8,3	serine (or cysteine) peptidase inhibitor, clade g, member 1
RN.9772	4,4	3,6	2,5	complement component 3a receptor 1
RN.6702	5,0	4,8	3,1	complement component 1, q subcomponent, beta polypeptide
RN.98333	4,2	4,6	4,0	complement component 2
RN.21393	3,7	6,5	5,7	coagulation factor x
				
**Cell Motility**				
RN.66513	5,8	7,2	5,8	arachidonate 12-lipoxygenase (predicted)
RN.6282	5,0	5,0	4,8	insulin-like growth factor 1
RN.2090	3,7	3,4	2,7	actin related protein 2/3 complex, subunit 1b
RN.14655	9,6	5,6	4,1	integrin alpha l
RN.95169	3,2	3,7	3,0	abi gene family, member 3
RN.101608	4,9	4,2	4,5	lymphocyte specific 1
RN.10139	10,9	12,8	10,1	chemokine (c-c motif) ligand 3
RN.49170	4,7	4,4	3,4	suppression of tumorigenicity 14
RN.42962	4,3	6,8	6,2	integrin beta 2
				
**Apoptosis**				
RN.66513	5,8	7,2	5,8	arachidonate 12-lipoxygenase (predicted)
RN.6282	5,0	5,0	4,8	insulin-like growth factor 1
RN.16195	3,7	4,0	3,2	caspase 11
RN.16643	4,8	4,2	3,1	fc receptor, igg, high affinity i
RN.101608	4,9	4,2	4,5	lymphocyte specific 1
RN.3176	4,2	4,7	3,8	lymphocyte antigen 86 (predicted)
RN.7110	6,0	4,9	4,2	receptor-interacting serine-threonine kinase 3
RN.18985	4,0	5,0	3,3	protein tyrosine phosphatase, non-receptor type 6
RN.42962	4,3	6,8	6,2	integrin beta 2
RN.10089	4,7	4,2	3,8	cyclin-dependent kinase inhibitor 1a
RN.90166	3,4	3,7	3,2	protein tyrosine phosphatase, receptor type, c
RN.10250	4,6	2,7	3,2	growth arrest and dna-damage-inducible 45 alpha
				
**Intracellular Signaling Cascade**				
RN.6282	5,0	5,0	4,8	insulin-like growth factor 1
RN.68084	6,3	4,3	7,7	b-cell leukemia/lymphoma 3 (predicted)
RN.780	7,0	3,9	4,1	alpha-2-macroglobulin
RN.35286	3,4	4,0	2,9	similar to ptpl1-associated rhogap 1 (predicted)
RN.138976	3,3	3,6	2,9	sh3-domain binding protein 2
RN.19450	19,4	25,9	18,5	adenylate cyclase 7
RN.38575	3,2	3,8	3,5	neutrophil cytosolic factor 1
RN.7110	6,0	4,9	4,2	receptor-interacting serine-threonine kinase 3
RN.18985	4,0	5,0	3,3	protein tyrosine phosphatase, non-receptor type 6
RN.11534	3,6	3,8	3,2	tyro protein tyrosine kinase binding protein
RN.10748	2,9	3,9	5,5	cd4 antigen
RN.48861	5,2	4,8	3,6	vav 1 oncogene
RN.40136	3,5	4,3	3,2	transforming growth factor, beta 1

*Only those genes and their corresponding functional classes found specifically enriched and significantly associated with epileptogenesis are shown (*P*-values ≤ 0.001, according to the Fisher Exact Test for functional enrichment analysis). Expression data is given as fold change from control, at the three time points analyzed after *SE *(D3 = three days after *SE*; D7 = seven days after SE; Chronic = immediately after the first spontaneous seizure).

**Table 2:** Regulatory signal transduction pathways and corresponding gene members found differentially expressed during epileptogenesis.

Signaling Pathway	D3 vs. CTRL		D7 vs. CTRL		CHRONIC vs. CTRL		D3, D7 and CHRONIC vs. CTRL	
			
	Up-regulated	Down-regulated	Up-regulated	Down-regulated	Up-regulated	Down-regulated	Up-regulated	Down-regulated
**MAPK**	*Tgfbr1, Acvr1c*	*Prkcc*	*Tgfb1*	-	*P38 Mapk, Fgf7, Rasa2*	*Map2k1*	*Cacna1g, Casp4, Gadd45a*	-
**Wnt**	*Plcb2*	*Prkcc*	-	-	-	-	-	-
**Notch**	-	*Hes5*	-	-	-	-	-	-
**TGF-beta**	*Tgfbr1, Tgfb1, Acvr1c*	-	*Tgfb1*	-	*Tgfb1*	-	*Tgfb1*	-
**Jak-STAT**	*Stat3, Jak3*	-	*Pik3r1, Ifngr1*	-	*Pik3r3*	-	*Csf2rb1*	-
**Calcium**	*Plcb2*	*Prkcc, Cckbr*	-	-	-	-	*Cacna1g*	-
**Phosphatidyl Inositol**	*Plcb2*	*Prkcc*	*Pik3r1*	-	*Pik3r3*	-	*Pik3c2b*	-
**mTOR**	*Igf1*		*Igf1*		*Igf1*		*Igf1*	

*D3 = three days post-SE; D7 = seven days post-*SE*; Chronic = immediately after the first spontaneous seizure; CTRL = saline-treated, time-matched control.

**Figure 3:**
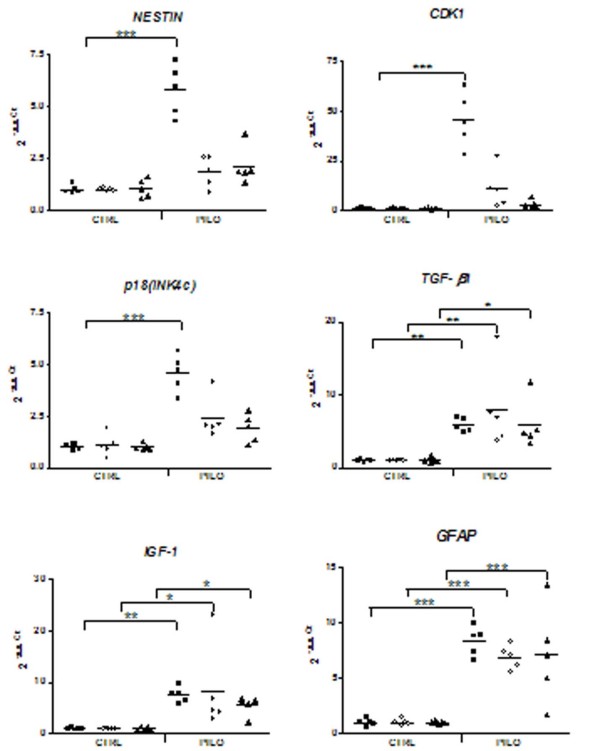
**Validation of microarray data by quantitative real-time PCR**. Relative gene expression in both control (CTRL) and pilocarpine (PILO) groups. Labels: (■) 3 days post-*SE*, (○) 7 days post-*SE*, and (▲) right after the first spontaneous seizure (Chronic). *P ≤ 0.05; **P ≤ 0.01; ***P ≤ 0.001.

Differential expression of the selected genes *Nestin, CDK1, p18(INK4c), TGF-β1, IGF-1 *and *GFAP *was quantified by real-time PCR in order to confirm the microarray results. Similar results were found with both techniques. Compared with the respective control groups, *Nestin, CDK1*, and *p18(INK4c) *genes were found significantly over-expressed only three days after SE. On the other hand, *TGF-β1, IGF-1*, and *GFAP *genes displayed significant variations in expression at all times analyzed after *SE *(Figure 3).

## Discussion

High throughput gene expression profiling data can be correlated with a certain pathological condition, subtype or phase of diseases. This genomic approach could facilitate the identification of molecular pathways and physiological processes involved in the development of complex multifactorial diseases such as epilepsy.

In animal models, differentially expressed genes have been reported in response to *SE *induced by kainic acid [11], pilocarpine [12,13], and by electric stimulation (Kindling) [14,15]. Despite the large amount of data already accumulated, a comparative meta-analysis is difficult to carry out due to the different experimental design and microarray platforms used in studies of such kind. The amount of genes/transcripts interrogated in most studies ranged from 1560 to 8000, corresponding to up to 25% of genes identified in the rat genome. Furthermore, the interval after *SE *in which gene expression has been evaluated varies from as early as one day, when many genes functioning in stress defense, inflammation, and cell death are regulated in response to the excitotoxic insult, to as late as 100 days post-*SE*, when gene expression reflects a neural tissue suffering from multiple events of spontaneous seizures, with frequency and intensity of seizures varying among testing animals.

One distinct aspect of the present study is that the experiments were designed to cover all possible changes in the transcriptome of hippocampal cells, using a microarray platform containing more than 34,000 oligonucleotide probes representing transcripts from all annotated rat genes to date. This whole-genome profiling was conducted at three close time-points during the course of epileptogenesis, according to the pilocarpine model of epilepsy: three and seven days post-*SE*, corresponding to early and mid term of the latent phase, and immediately after the first spontaneous seizure which corresponds to the onset of the chronic phase. The major aim was to uncover genes that might be involved in development of epilepsy and not solely correlated with *SE *or seizures. However, given that this strategy was applied to the whole hippocampus, information regarding particular cell types and subregions within the hippocampus in which abnormal gene expression occurs is not available and should be further investigated.

Nonetheless, since pilocarpine-treated animals generally return to normal behavior within three days post-*SE*, a molecular analysis at this time point should capture the early events leading to epilepsy with minimal contamination by genes regulated in response to the chemical treatment *per se *or as a consequence of tissue damage. However, many of the genes found differentially expressed three days post-*SE *were related to stress response including participation in defense mechanisms and wound healing. This result is in agreement with a previous study by Becker et al., 2003 [12] reporting, three days post-*SE*, a high proportion of up-regulated genes in the dentate gyrus and CA1 regions of the hippocampus being associated with cellular stress and injury. However, under the same experimental condition, we also found a large group of genes functioning in cell proliferation and migration, supporting that neurogenesis is triggered early in latent phase.

In agreement with this assumption, quantitative real-time PCR analysis of cell cycle genes confirmed hyper-expression of *CDK1*, a gene regulating the G1 to S and G2 to M transition of the cell cycle, and *Nestin*, a marker of neural stem cells and neural progenitor cells. However, expression of the cell cycle inhibitor *p18(INK4c) *was paradoxically enhanced after *SE *and coincide with the peak of *CDK1 *and *Nestin *expression at day three post-*SE*. No significant differences in the expression of these genes were observed seven days post-*SE *or after the first spontaneous seizure. Recent studies have reported that members of the cyclin dependent kinase family are up-regulated during epileptogenesis [30]. However, the hyper-expression of the cell cycle inhibitor *p18(INK4c) *found after *SE *had not been previously reported. This finding suggests that the proliferative stage of neurogenesis in the hippocampus of adult rats may be inhibited by such activation of *p18(INK4c)*.

Moreover, when examining gene expression profiles in animals that had experienced only one spontaneous seizure after *SE*, a time by when all essential changes for the installation of epilepsy are expected to be manifested in the central nervous system, there was a primarily up-regulation of genes functioning in protein expression and post-translational processing, including many transcription factors, kinases and other transferases, along with genes regulating cell differentiation such as *FOXD3 *and *RUNX1*. These two genes are of particular interest since they encode transcription factors involved in maintenance of embryonic stem cell pluripotency and proliferation of neural progenitors, respectively [31,32]. Since commitment of stem cells to the neuronal lineage is associated with repression of *FOXD3 *expression [33], it is reasonable to assume that its hyper-expression could be implicated in inhibition of neuroprogenitor differentiation. This observation is in line with a marked repression of genes regulating cell fate, development, and morphogenesis such *as LDB2, BMP3*, and *MAP2K1 *detected in pilocarpine-treated animals at the beginning of the chronic phase.

Altogether, these findings suggest that neurogenesis may be impaired by different mechanisms throughout the period corresponding to the latent to chronic phase transition of the pilocarpine model. Indeed, anomalous neurogenesis has been implicated in the development of epilepsy [34,35]. Prolonged seizures are known to stimulate neurogenesis in the dentate gyrus, although new born neurons display abnormal apical dendritic morphology, and the ensuing plasticity of the hippocampal network can lead to aberrant neuronal connections associated with epileptogenesis [36,37]. Furthermore, fate decision of proliferating neuronal progenitors in the hippocampus has been reported to be impaired after intra-hippocampal injection of kainic acid, favoring astrogliosis [38]. Prolonged seizures have also been reported to induce aberrant migration of newborn neurons to the hilus and inner molecular layer of the dentate gyrus, contributing to the formation of a hyperexcitable circuitry [39,35].

Another intriguing result that emerged from the comparative analysis of genes differentially expressed at each of the three time points post-*SE *was the identification of a group of 128 genes continually up-regulated throughout the course of epileptogenesis. This finding was somewhat unexpected since cellular activity in response to its microenvironment stimuli is dynamically regulated through transient and specific changes in gene expression. One possible interpretation is that the *SE *may cause stable alterations in the microenvironment, exposing the remaining cells to nonstandard cues. Alternatively, the nature of cell population within the hippocampus may be shifted after *SE*, accommodating a greater proportion of activated microglial cells and other cells of abnormal phenotype expressing a different repertoire of genes.

Consistent with the literature, a functional classification of these 128 up-regulated genes indicated that most of them are involved in signaling cascades, extracellular matrix remodeling and cell motility, apoptosis, and immune response, which are processes known to be associated with epileptic seizures. In particular, the high proportion of immune response genes and the activation of genes from the p38 MAPK signaling pathway, some of which confirmed by real-time PCR, are in agreement with a chronic activation of microglial cells [40]. Furthermore, up-regulation of proinflammatory genes in both cortex and hippocampus of patients with TLE have been recently reported [41].

Based on experimental and clinical evidences, inflammation and neurogenesis seem to be common factors contributing to the development of epilepsy. Since microglia activation suppresses hippocampal neurogenesis in adult brain [42], modulation of genes and pathways connecting both processes should have an impact on epileptogenesis. Whether some of the genes hereby found regulated during epileptogenesis should fall into this category of relevance remains to be elucidated. Interestingly, the steady up-regulation of the inflammatory peptide TGF-beta1 detected throughout epileptogenesis might be one of such factors contributing to an anomalous neurogenesis. This hypothesis is reinforced by a recent report by Cacheaux et al., 2009 [43], describing involvement of the TGF-beta pathway in the development of epilepsy caused by brain injury.

## Conclusions

In summary, our whole-genome screening indicate remarkable changes in the rat transcriptome along the latent to chronic phase transition of the pilocarpine model of epilepsy. Over a thousand genes had their expression altered after *SE*, most of which displaying an up-regulation, and their functional classification provide a molecular portrait of the epileptogenic process. There was a consistent regulation of genes functioning in neurogenesis, apoptosis, immune response, and intracellular signal transduction, some of which with parallel expression patterns in patients with epilepsy, strengthening the link between these processes and development of TLE. Most differentially expressed genes identified are novel and could now be further tested to provide experimental and clinical evidences of functional relevance to epileptogenesis. In particular, genes mediating inflammation and neurogenesis such as those from the TGF-beta signaling pathway are of particular interest and shall be pursued as targets for molecular therapy.

## Authors' contributions

OKO conceived the study, conducted experiments, discussed results and wrote the manuscript. EAC conceived the study, discussed results and revised the manuscript. AVS & FAS discussed results and revised the manuscript. L.J., F.M.B., and A.P.P conducted experiments, discussed results and help writing the manuscript. All authors have read and approved the final manuscript.

## References

[B1] Sander JW The epidemiology of epilepsy revisited Curr Opin Neurol 2003 16 165 10 10.1097/00019052-200304000-00008 12644744

[B2] Kwan P Sander JW The natural history of epilepsy: an epidemiological view J Neurol Neurosurg Psychiatry 2004 75 376 381 10.1136/jnnp.2004.045690 PMC1738749 15377680

[B3] Engel J Jr Clinical neurophysiology, neuroimaging, and the surgical treatment of epilepsy Curr Opin Neurol Neurosurg 1993 6 Suppl 2 240 249 8481567

[B4] Dalby NO Mody I The process of epileptogenesis: a pathophysiological approach Curr Opin Neurol 2001 14 187 192 10.1097/00019052-200104000-00009 11262734

[B5] Arion D Sabatini M Unger T Pastor J Alonso-Nanclares L Ballesteros-Yanez I Garcia Sola R Munoz A Mirnics K DeFelipe J Correlation of transcriptome profile with electrical activity in temporal lobe epilepsy Neurobiol Dis 2006 22 374 387 10.1016/j.nbd.2005.12.012 16480884

[B6] Jamali S Bartolomei F Robaglia-Schlupp A Massacrier A Peragut JC Regis J Dufour H Ravid R Roll P Pereira S Royer B Roeckel-Trevisiol N Fontaine M Guye M Boucraut J Chauvel P Cau P Szepetowski P Large-scale expression study of human mesial temporal lobe epilepsy: evidence for dysregulation of the neurotransmission and complement systems in the entorhinal cortex Brain 2006 129 625 641 10.1093/brain/awl001 16399808

[B7] Berkovic SF Dibbens LM Oshlack A Silver JD Katerelos M Vears DF Lüllmann-Rauch R Blanz J Zhang KW Stankovich J Kalnins RM Dowling JP Andermann E Andermann F Faldini E D'Hooge R Vadlamudi L Macdonell RA Hodgson BL Bayly MA Savige J Mulley JC Smyth GK Power DA Saftig P Bahlo M Array-based gene discovery with three unrelated subjects shows SCARB2/LIMP-2 deficiency causes myoclonus epilepsy and glomerulosclerosis Am J Hum Genet 2008 82 673 684 10.1016/j.ajhg.2007.12.019 18308289 PMC2427287

[B8] Helbig I Matigian NA Vadlamudi L Lawrence KM Bayly MA Bain SM Diyagama D Scheffer IE Mulley JC Holloway AJ Dibbens LM Berkovic SF Hayward NK Gene expression analysis in absence epilepsy using a monozygotic twin design Epilepsia 2008 49 1546 1554 10.1111/j.1528-1167.2008.01630.x 18435749

[B9] Xi ZQ Xiao F Yuan J Wang XF Wang L Quan FY Liu GW Gene expression analysis on anterior temporal neocortex of patients with intractable epilepsy Synapse 2009 63 1017 1028 10.1002/syn.20681 19623530

[B10] Scharfman HE Gray WP Relevance of seizure-induced neurogenesis in animal models of epilepsy to the etiology of temporal lobe epilepsy Epilepsia 2007 48 33 41 10.1111/j.1528-1167.2007.01065.x 17571351 PMC2504501

[B11] Hunsberger JG Bennett AH Selvanayagam E Duman RS Newton SS Gene profiling the response to kainic acid induced seizures Brain Res Mol 2005 141 95 112 10.1016/j.molbrainres.2005.08.005 16165245

[B12] Becker AJ Chen J Zien A Sochivko D Normann S Schramm J Elger CE Wiestler OD Blümcke I Correlated stage- and subfield-associated hippocampal gene expression patterns in experimental and human temporal lobe epilepsy Eur J Neurosci 2003 18 2792 802 10.1111/j.1460-9568.2003.02993.x 14656328

[B13] Elliott RC Miles MF Lowenstein DH Overlapping microarray profiles of dentate gyrus gene expression during development- and epilepsy-associated neurogenesis and axon outgrowth J Neurosci 2003 23 2218 2227 12657681 10.1523/JNEUROSCI.23-06-02218.2003 PMC6742005

[B14] Lukasiuk K Kontula L Pitkanen A cDNA profiling of epileptogenesis in the rat brain Eur J Neurosci 2003 17 271 279 10.1046/j.1460-9568.2003.02461.x 12542663

[B15] Gorter JA van Vliet EA Aronica E Breit T Rauwerda H Lopes da Silva FH Wadman WJ Potential new antiepileptogenic targets indicated by microarray analysis in a rat model for temporal lobe epilepsy J Neurosci 2006 26 11083 11110 10.1523/JNEUROSCI.2766-06.2006 17065450 PMC6674659

[B16] Turski L Ikonomidou C Turski WA Bortolotto ZA Cavalheiro EA Cholinergic mechanisms and epileptogenesis. The seizures induced by pilocarpine: a novel experimental model of intractable epilepsy Synapse 1989 3 154 171 10.1002/syn.890030207 2648633

[B17] Cavalheiro EA Leite JP Bortolotto ZA Turski WA Ikonomidou C Turski L Long-term effects of pilocarpine in rats: structural damage of the brain triggers kindling and spontaneous recurrent seizures Epilepsia 1991 32 778 782 10.1111/j.1528-1157.1991.tb05533.x 1743148

[B18] Curia G Longo D Biagini G Jones RS Avoli M The pilocarpine model of temporal lobe epilepsy J Neurosci Methods 2008 172 143 157 10.1016/j.jneumeth.2008.04.019 18550176 PMC2518220

[B19] Turski WA Cavalheiro EA Schwarz M Czuczwar SJ Kleinrok Z Turski L Limbic seizures produced by pilocarpine in rats: behavioural, electroencephalographic and neuropathological study Behav Brain Res 1983 9 315 335 10.1016/0166-4328(83)90136-5 6639740

[B20] Leite JP Bortolotto ZA Cavalheiro EA Spontaneous recurrent seizures in rats: an experimental model of partial epilepsy Neurosci Biobehav Rev 1990 14 511 517 10.1016/S0149-7634(05)80076-4 2287490

[B21] Eisen MB Spellman PT Brown PO Botstein D Cluster analysis and display of genome-wide expression patterns Proc Natl Acad Sci 1998 95 14863 14868 10.1073/pnas.95.25.14863 9843981 PMC24541

[B22] Dennis G Jr Sherman BT Hosack DA Yang J Gao W Lane HC Lempicki RA DAVID: Database for Annotation, Visualization, and Integrated Discovery Genome Biol 2003 4 5 P3 10.1186/gb-2003-4-5-p3 12734009 PMC193660

[B23] Kanehisa M Goto S KEGG: kyoto encyclopedia of genes and genomes Nucleic Acids Res 2000 28 27 30 10.1093/nar/28.1.27 10592173 PMC102409

[B24] Ashburner M Ball CA Blake JA Botstein D Butler H Cherry JM Davis AP Dolinski K Dwight SS Eppig JT Harris MA Hill DP Issel-Tarver L Kasarskis A Lewis S Matese JC Richardson JE Ringwald M Rubin GM Sherlock G Gene ontology: tool for the unification of biology. The Gene Ontology Consortium Nat Genet 2000 25 25 29 10.1038/75556 10802651 PMC3037419

[B25] Wu X Yoo S Wrathall JR Real-time quantitative PCR analysis of temporal-spatial alterations in gene expression after spinal cord contusion J Neurochemistry 2005 93 943 952 10.1111/j.1471-4159.2005.03078.x 15857397

[B26] Turner KJ McIntyre BS Phillips SL Barlow NJ Bowman CJ Foster PMD Altered gene expression during rat wolffian duct development in response to in utero exposure to the antiandrogen linuron Toxicol Sci 2003 74 114 128 10.1093/toxsci/kfg096 12730624

[B27] Wang JM Johnston PB Ball BG Brinton RD The neurosteroid allopregnanolone promotes proliferation of rodent and human neural progenitor cells and regulates cell-cycle gene and protein expression J Neurosci 2005 19 4706 4718 10.1523/JNEUROSCI.4520-04.2005 PMC6724768 15888646

[B28] Ji JF Dheen ST Kumar SD He BP Tay SSW Expressions of cytokines and chemokines in the dorsal motor nucleus of the vagus nerve after right vagotomy Mol Brain Res 2005 142 47 57 10.1016/j.molbrainres.2005.09.017 16260063

[B29] Mandhan P Beasley S Hale T Ellmers L Roake J Sullivan M Sonic hedgehog expression in the development of hindgut in ETU-exposed fetal rats Pediatr Surg Int 2006 22 31 36 10.1007/s00383-005-1575-6 16369776

[B30] Murashima YL Suzuki J Yoshii M Cell cycle reentry and cell proliferation as candidates for the seizure predispositions in the hippocampus of EL mouse brain Epilepsia 2007 48 Suppl 5 119 125 10.1111/j.1528-1167.2007.01299.x 17910591

[B31] Theriault FM Nuthall HN Dong Z Lo R Barnabe-Heider F Miller FD Stifani S Role for Runx1 in the proliferation and neuronal differentiation of selected progenitor cells in the mammalian nervous system J Neurosci 2005 25 2050 2061 10.1523/JNEUROSCI.5108-04.2005 15728845 PMC6726063

[B32] Pan G Li J Zhou Y Zheng H Pei D A negative feedback loop of transcription factors that controls stem cell pluripotency and self-renewal FASEB J 2006 20 1730 1732 10.1096/fj.05-5543fje 16790525

[B33] Perry P Sauer S Billon N Richardson WD Spivakov M Warnes G Livesey FJ Merkenschlager M Fisher AG Azuara V A dynamic switch in the replication timing of key regulator genes in embryonic stem cells upon neural induction Cell Cycle 2004 3 1645 1650 15611653

[B34] Parent JM Yu TW Leibowitz RT Geschwind DH Sloviter RS Lowenstein DH Dentate granule cell neurogenesis is increased by seizures and contributes to aberrant network reorganization in the adult rat hippocampus J Neurosci 1997 17 3727 3738 9133393 10.1523/JNEUROSCI.17-10-03727.1997 PMC6573703

[B35] Parent JM Elliott RC Pleasure SJ Barbaro NM Lowenstein DH Aberrant seizure-induced neurogenesis in experimental temporal lobe epilepsy Ann Neurol 2006 59 81 91 10.1002/ana.20699 16261566

[B36] Arisi GM Garcia-Cairasco N Doublecortin-positive newly born granule cells of hippocampus have abnormal apical dendritic morphology in the pilocarpine model of temporal lobe epilepsy Brain Res 2007 1165 126 134 10.1016/j.brainres.2007.06.037 17662262

[B37] Parent JM Adult neurogenesis in the intact and epileptic dentate gyrus Prog Brain Res 2007 163 529 540 full_text 17765736 10.1016/S0079-6123(07)63028-3

[B38] Ledergerber D Fritschy JM Kralic JE Impairment of dentate gyrus neuronal progenitor cell differentiation in a mouse model of temporal lobe epilepsy Exp Neurol 2006 199 130 142 10.1016/j.expneurol.2006.02.010 16624297

[B39] Scharfman HE Goodman JH Sollas AL Granule-like neurons at the hilar/CA3 border after status epilepticus and their synchrony with area CA3 pyramidal cells: functional implications of seizure-induced neurogenesis J Neurosci 2000 20 6144 6158 10934264 10.1523/JNEUROSCI.20-16-06144.2000 PMC6772593

[B40] Choi YS Cho HY Hoyt KR Naegele JR Obrietan K IGF-1 receptor-mediated ERK/MAPK signaling couples status epilepticus to progenitor cell proliferation in the subgranular layer of the dentate gyrus Glia 2008 56 791 800 10.1002/glia.20653 18338791 PMC4152854

[B41] Argañaraz GA Konno AC Perosa SR Santiago JF Boim MA Vidotti DB Varella PP Costa LG Canzian M Porcionatto MA Yacubian EM Sakamoto AC Carrete H Jr Centeno RS Amado D Cavalheiro EA Junior JA Mazzacoratti MG The renin-angiotensin system is upregulated in the cortex and hippocampus of patients with temporal lobe epilepsy related to mesial temporal sclerosis Epilepsia 2008 49 1348 1357 10.1111/j.1528-1167.2008.01581.x 18363708

[B42] Ekdahl CT Claasen JH Bonde S Kokaia Z Lindvall O Inflammation is detrimental for neurogenesis in adult brain Proc Natl Acad Sci 2003 100 13632 13637 10.1073/pnas.2234031100 14581618 PMC263865

[B43] Cacheaux LP Ivens S David Y Lakhter AJ Bar-Klein G Shapira M Heinemann U Friedman A Kaufer D Transcriptome profiling reveals TGF-beta signaling involvement in epileptogenesis J Neurosci 2009 29 8927 8935 10.1523/JNEUROSCI.0430-09.2009 19605630 PMC2875073

